# Dr. E. B. Gardette and the American Society of Dental Surgeons

**Published:** 1841-12

**Authors:** 


*Dr. E. B. Gardette and the American Society of Dental Surgeons.*—We put ourself to some inconvenience to publish the following communication from Dr. Gardette. We really think that he has taken an improper view of our conduct in the matter complained of, and in proof of this, we refer our readers to his own statement. It will be seen that Dr. Gardette was regularly elected a Vice-president of the American Society of Dental Surgeons, and regularly notified of the fact. At the same time he was informed that the proceedings of the Society, and of course his own election, would be published. Dr. Gardette did not then decline the office, nor object to the publication of his name, but, as he states, he "delayed answering the letter (informing him of his election) until he should have received the Journal, and duly considered the character of the meeting and proceedings referred to." Of course, then Dr. Gardette expected to see his name announced as one of the vice-presidents of the society; he would have been surprised and doubtless offended, if it had not been so; and yet he declares that his mind was not made up about even the character of the meeting in question! Unquestionably Dr. Gardette's silence was a tacit acceptance of the office, and his subsequent letter could only be considered as a *resignation.* This, the society accepted as soon as it was tendered, but they did not think it necessary or important to publish his letter.

We have no unkind feelings towards Dr. Gardette, and if he thinks the publication of his letter necessary for his vindication, we are willing to oblige him, but we think he has had no reason for charging us with any improper conduct in the matter.—*Balt. Ed.*

*To* C. A. Harris, M. D. *Corresponding Secretary of the American Society of Dental Surgeons.*

Dear Sir: *Philadelphia, November,* 1841.

It is with some reluctance, and yet as an act of justice to myself, that I adopt this public mode of expressing my views of the course pursued towards me by "*The American Society of Dental Surgeons,*" and yourself, as its corresponding secretary. I have been, I think, very patient in affording abundant time for an act of ordinary justice and civility at the hands of the society or its official organ, by giving *the same publicity to my non-acceptance of the office of vice-president, as was extended by them to my unsolicited election to that post.* I trust therefore you will excuse me for requesting the publication of your official communication to me dated August, 1840, with my reply, and this explanation of the circumstances under which I do so.

"*The American Journal of Dental Science,*" of which you were an editor, has given to the public the proceedings of your society in New York, and my name being there mentioned as a vice-president, I naturally supposed the same journal would have announced the fact of my non-acceptance. This however was not the case, and I then looked to the next meeting of "*The American Society of Dental Surgeons,*" which took place in August, 1841, as the proper time for action in the matter, and expected of course that my refusal would be found in the then published proceedings. In this expectation I have again been disappointed: The first number of "*The American Journal and Library of Dental Science*," just received, contains "*Extracts from the Transactions*," of the second meeting of the society held in August last, and also the new organization of its officers; and although my name, it is true, does not again appear, I find no notice of the fact that I had *voluntarily* declined the office of vice-president immediately on my election. This simple statement would at least have made it appear to my friends abroad, that I was not a candidate for such distinction at the second election.

I take this occasion to say publicly that I never had the pleasure of being present at a meeting of the above named society, nor at the convention in New York, for its organization, and that I never authorized any one to represent me on either occasion.

I am very respectfully, your obedient servant, E. B. GARDETTE.

[COPY.]

DEAR SIR: *Baltimore, August,* 1840.

The "American Society of Dental Surgeons," at a meeting held in New York, August 18, 19 and 20th, after the adoption of a constitution and by-laws, elected you one of its vice-presidents. The proceedings of the society together with its constitution and by-laws, you will receive in the next No. of the "American Journal of Dental Science," which will be out in a few days. With sentiments of respect, &c.

C. A. HARRIS. *Cor. Sec. of the Society.*

DR. E. B. GARDETTE.

[REPLY.]

DEAR SIR: *Philadelphia, September,* 1840.

Your favour of August twenty-seventh, was duly received, announcing that the "American Society of Dental Surgeons," at a meeting held in New York, had elected me one of its vice-presidents. Your accompanying promise, that the proceedings of the society together with its constitution and by-laws would be published in the coming number of "The American Journal of Dental Science," induced me to delay answering your letter, until I should have received the journal, and duly considered the character of the meeting and proceedings referred to.

I now beg respectfully to tender to the society, through you as its proper organ, my resignation or rather *non-acceptance* of the office of vice-president, and the privilege of an active member of the society with which it has honoured me without my consent.

My thanks are due to you individually for the polite interest you have manifested that I should take an active part in the organization of this society; but being unable to attend the meeting at New York, I had hoped that the character of my reply by letter to you in June last, as well as my views previously expressed in conversation at my house, would have led you to conclude, that a society, formed as this has been, could never receive my sanction, much less induce me to accept a prominent post in it.

You will be good enough to communicate to the society, my non-acceptance of the office of vice-president, and of an active member.

I am very respectfully yours, &c. E. B. GARDETTE.

*To* DR. C. A. HARRIS, *Cor. Sec. Am. Soc. of Dental Surgeons.*

